# Characterization of the Polycaprolactone Melt Crystallization: Complementary Optical Microscopy, DSC, and AFM Studies

**DOI:** 10.1155/2014/720157

**Published:** 2014-01-09

**Authors:** V. Speranza, A. Sorrentino, F. De Santis, R. Pantani

**Affiliations:** ^1^Department of Industrial Engineering (DIIN), University of Salerno, Via Giovanni Paolo II, 84084 Fisciano, Italy; ^2^Institute for Composite and Biomedical Materials (IMCB), National Research Council (CNR), Piazzale Enrico Fermi 1, 80055 Portici, Italy

## Abstract

The first stages of the crystallization of polycaprolactone (PCL) were studied using several techniques. The crystallization exotherms measured by differential scanning calorimetry (DSC) were analyzed and compared with results obtained by polarized optical microscopy (POM), rheology, and atomic force microscope (AFM). The experimental results suggest a strong influence of the observation scale. In particular, the AFM, even if limited on time scale, appears to be the most sensitive technique to detect the first stages of crystallization. On the contrary, at least in the case analysed in this work, rheology appears to be the least sensitive technique. DSC and POM provide closer results. This suggests that the definition of induction time in the polymer crystallization is a vague concept that, in any case, requires the definition of the technique used for its characterization.

## 1. Introduction

The phenomenon of crystallization of polymers has been studied since the start of the macromolecular science. Despite the great efforts spent by the scientific community on this topic, the formation of crystalline structures between high-entangled polymer chains is still debated.

A complete investigation of the crystallization process requires the use of several analytical techniques, since a single technique does not supply sufficient understanding of the complex multiscale transformation. Heat transition involved in the process is typically measured by differential scanning calorimeter (DSC-DTA), structures formation is generally monitored by microscopy (optical, AFM, TEM, or SEM), and order alteration is observed by X-ray diffraction and infrared spectroscopy, whereas changes in flow properties are commonly measured with rheological tests [1, 2]. However, an accurate comparison of the quantities measured on individual instruments is not straightforward, since not only the scale but also the samples conditions are always different.

The application of atomic force microscopy (AFM) to polymer system is often devoted to the observation of a solidified sample after quenching the crystal structure, as a static subsequent step. Most dynamic hot stage studies of morphological development in situ have been performed using optical microscopy, with its limited resolution.

Recently, the possibility to follow real-time dynamics of processes at nanoscale, even if limited on time scale, is opened to AFM. Since the initial in situ observations of the polymer crystallization reported by Pearce and Vancso [3–5] and by Schultz and Miles [6] several additional real-time AFM studies were reported [7–14]. Some of these studies were performed by controlling the temperature of the sample [10, 11] and sometimes the temperature of the AFM tip [13, 14].

Pearce and Vancso [3, 4] first reported the consistency of in situ AFM results with conventional optical microscopy results. They found that growth rates of individual lamellae agree with results obtained at lower magnification by optical microscopy. Similar growth rates were observed at the growth front by Hobbs et al. [9] whereas observations at the scale of the lamella revealed for the first time that dominant lamellae do not grow at a constant rate. Lamellae grow at sporadic or constant rates depending on the presence or not of surrounding lamellae competing at the growth front [10, 11, 14].

In situ AFM studies gave the opportunity to observe developing morphologies at a lamellar scale, from the generation of the primary stable nucleus [8] to the development of dominant lamellae, followed by the birth of branching lamellae that splay, leading to the spherulitic morphology [4, 6, 8] with, finally, impingement of adjacent spherulites [5].

These observations highlighted the roughness of the growth front, seen as being smooth at the larger scale of optical microscopy [9].

In particular for the investigation of material transformations that are associated with changes in rheological and thermal properties, such as melting and crystallization, an accurate comparison between these techniques is crucial. In the literature, a few attempts for combining morphology, calorimetry, and rheology can be found [15].

Martins et al. [16] developed a shear differential thermal analyzer that allowed for applying controlled shear pulses during isothermal or nonisothermal solidification of polymers. Hereto, a capillary rheometer and a differential thermal analyzer were combined into a single device. The instrument has two capillaries, one for the investigated sample and one for a reference material, which are placed inside two separate identical ovens.

Nagatake et al. [17] modified a commercial rheometer by adding two thermocouples on the base plate, which allowed the measurement of differences between sample and base plate temperatures.

In this paper, the investigation of the morphology development of melt-grown crystal lamellae of polycaprolactone by real-time hot stage AFM is reported. The morphological insights are also investigated by using polarized optical microscopy (POM), differential scanning calorimetry, and rheology tests.

## 2. Experimental

### 2.1. Material

The polymer investigated in this work is polycaprolactone (*M*_*n*_ 70000–90000 by GPC, *M*_*w*_/*M*_*n*_ < 2, density 1.145 g/mL at 25°C), supplied by Sigma Aldrich.

### 2.2. Differential Scanning Calorimetry

A differential scanning calorimeter DSC 822 from Mettler Toledo Inc. was used for determination and measurement of the calorimetric behavior of PCL. The calibration of the temperature was done with the extrapolated onset temperature of the phase transition of indium. The samples with a weight of about 10 mg were put into an aluminum pan. All the experiments were conducted in nitrogen (flow rate 50 mL/min), in order to prevent oxidative degradation.

### 2.3. Optical Microscopy

A PCL thin film, of about 100 *μ*m, is sandwiched between two cover glasses and this is observed in light transmission using an optical Olympus BX51 Microscope equipped with digital camera. To apply the same thermal protocol to the glass/polymer system a special calorimeter (Linkam DSC600, calibrated with indium, cooled with liquid nitrogen, and purged with nitrogen [18]) was adopted for the experiments. The morphology and the isothermal growth of the crystalline structures were observed using the microscope coupled to the Linkam DSC600 during the calorimetric tests.

### 2.4. Atomic Force Microscopy

In order to characterize the local morphology of the samples a Veeco Dimension 3100 AFM with Nanoscope III controller was adopted. All the sample images were kept using the tapping mode in order to minimize the interaction with the sample surface. Two tests were conducted with the same thermal history, but analyzing different surfaces: one test explored a square surface having a side of 10 *μ*m and the other one a square surface having a side of 30 *μ*m. In both experiments, the scan rate was 2 Hz and 256 sample lines. The Linkam hot stage THMS600 is used for conditioning the sample during the in situ AFM characterization. The equilibrium melting temperature of PCL is relatively low, about 60°C, so that it was possible to perform the AFM acquisition without altering the linearity of the AFM piezo.

### 2.5. Rheological Tests

Rheological experiments were performed using a stress controlled rotational rheometer (HAAKE RheoScope) equipped with 25 mm parallel plates and using a gap thickness of 0.1 mm. To follow both the cooling process and the isothermal crystallization, the evolution of the mechanical properties has been monitored by an oscillatory test at the constant stress, *σ* = 100 Pa, and constant frequency, *ω* = 0.1 rad/sec. The choice of these values is crucial in order to reduce, as much as possible, the disturbance during the crystallization and ensure that the whole crystallization process occurs in the range of sensitivity of the equipment. To verify whether these parameters could affect the experimental results, tests with stress values ranging from 1 to 1000 Pa have been performed.

Optical observations were made directly in the rheometer by a CCD camera (HAAKE RheoScope) equipped with a 5x magnification objective.

### 2.6. Methods

The same thermal history has been applied to all the experiments. In particular, an annealing treatment has been conducted at *T* = 120°C for 15 min. The cooling step from the annealing temperature (*T*_*a*_) to the crystallization temperature (*T*_*c*_) has been performed in two steps. The first step from *T*_*a*_ to the melting temperature *T*_*m*_ = 60°C was performed at a rate compatible with the limitations of the different experimental techniques (−10°C min^−1^ for DSC, AFM, and POM; −3°C min^−1^ for rheological tests) whereas the second step from *T*_*m*_ to *T*_*c*_ was performed at a rate of −0.5°C min^−1^. The temperature profile has been verified by an independent thermocouple placed inside the molten sample. The temperature sensors of the rheometer, the calorimeter, and the hot stage were carefully calibrated to minimize temperature differences among the instruments.

## 3. Results and Discussion

### 3.1. DSC Experiments

**Figure 1:**
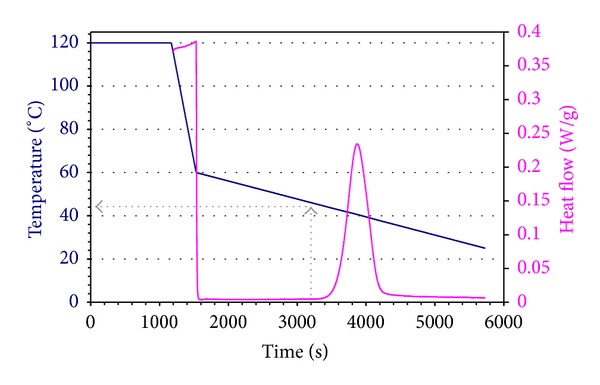
Temperature and calorimetric evolution during preliminary DSC test.

A preliminary calorimetric test was performed in order to determine the relevant temperatures for the crystallization phenomena of PCL. The sample was heated to 120°C at 30 C/min, kept at this temperature for 15 minutes, and then cooled down to 60°C at −10 C/min and from 60 to 25°C at −0.5 C/min. The result of this test is reported in Figure 1.

The calorimetric plot evidences an exothermic peak due to the crystallization of PCL, which starts at 44°C, has a maximum at 39°C, and ends at 34°C.

**Figure 2:**
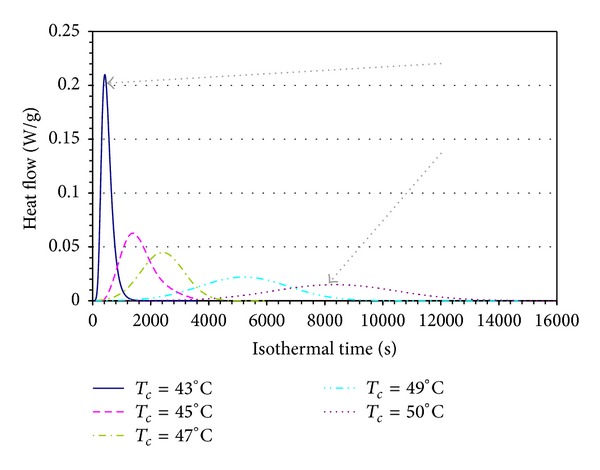
Experimental DSC tests.

A series of isothermal crystallization tests were thus carried out at temperatures between 50°C and 43°C. The corresponding exothermic peaks are reported in Figure 2 as a function of the time of the isothermal step. As expected, by increasing the crystallization temperature the peaks decrease in height and spread along the time axis. The area delimited by the peaks, characteristic of the heat released during the crystallization, is almost constant for all the experiments.

The crystallization heat, obtained by the measurement of the area under the exothermic peak, can be transformed into the relative degree of crystallinity (*α*_DSC_(*t*)) by the division of the heat that develops at each crystallization time *t* (Δ*H*_*t*_) by the total area under the exothermic peak, that is, the total heat (Δ*H*_0_) generated up to the complete crystallization:

(1) $$\begin{array}{c} \alpha_{\text{DSC}}\left(t\right)=\frac{\int_{0}^{t}\left(dH/dt\right)dt}{\int_{0}^{\infty }\left(dH/dt\right)dt}=\frac{\Delta H_{t}}{\Delta H_{0}}. \end{array}$$

**Figure 3:**
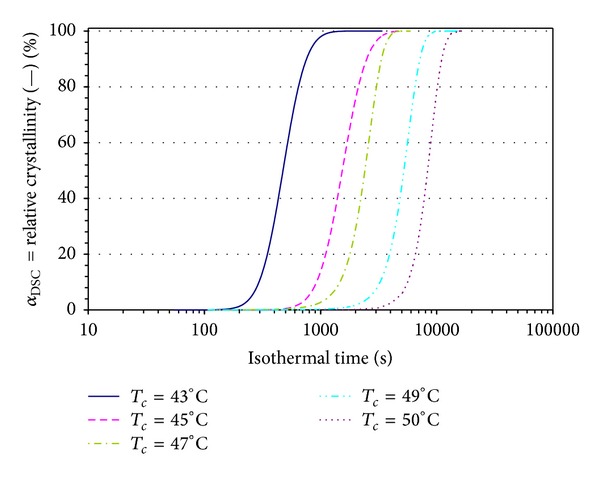
Evolution of relative crystallinity (*α*_DSC_(*t*) defined by (1)) in isothermal crystallization.

Figure 3 displays the development of *α*_DSC_ as a function of the isothermal time at several crystallization temperatures. A shift of the curves toward lower times and an increment of the slope of their linear portion can be seen as the crystallization temperature becomes lower. This implies that a higher undercooling degree leads to a higher crystallization rate.

An approximation of the crystallization rate can be made by the overall crystallization rate (1/*t*_0.5_), where *t*_0.5_ (half the crystallization time) is the time at which *α*_DSC_(*t*) approaches the value of 50%. This parameter is proportional to both the primary nucleation rate and the crystal/spherulite growth [19]. The induction time (*t*_0_), that is, the time at which *α*_DSC_(*t*) starts to increase, is also clear from Figure 3. As expected, t_0_ increases exponentially with the crystallization temperature.

### 3.2. POM Experiments

The morphology developing during the crystallization of PCL was investigated by polarized optical microscopy (POM). The micrographs were acquired at suitable time intervals with crossed polarizer during the same isothermal tests performed during the DSC tests.

**Figure 4:**
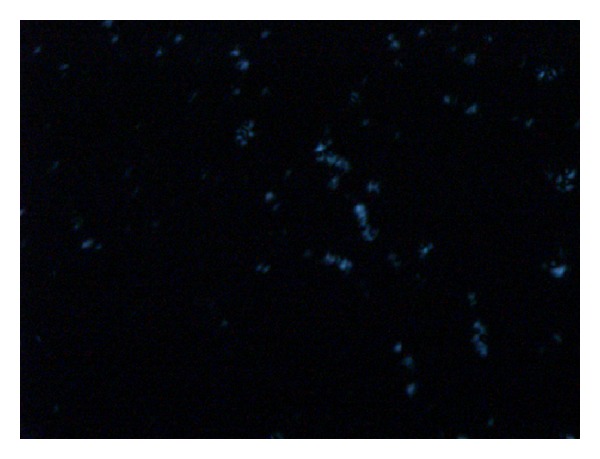
Optical image of PCL at the start of the isothermal crystallization (hot stage temperature = 45°C), 50x (image dimensions: 175 × 131.2 *μ*m).

**Figure 5:**
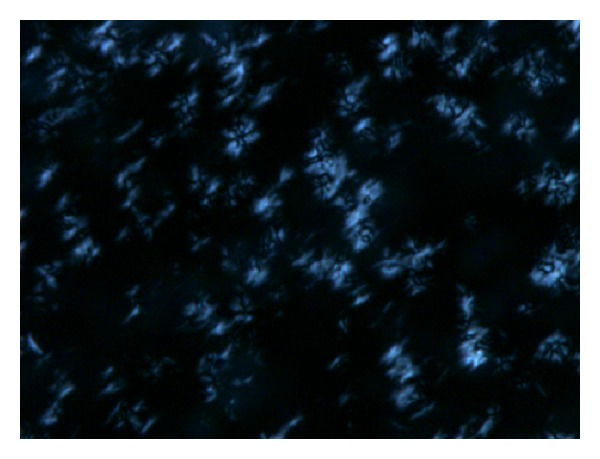
Optical image of PCL after 1800 seconds of isothermal crystallization (hot stage temperature = 45°C), 50x (image dimensions: 175 × 131.2 *μ*m).

**Figure 6:**
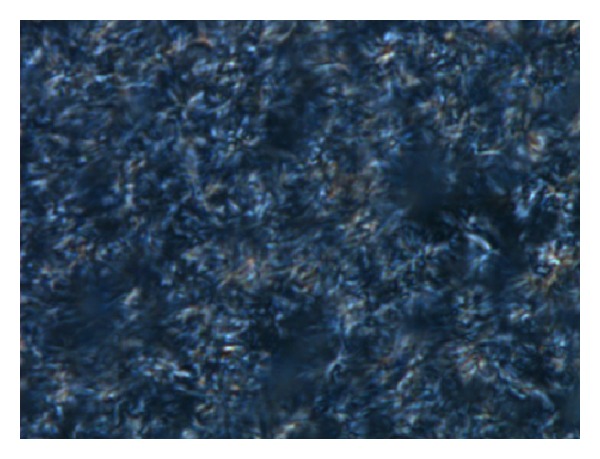
Optical image of PCL after 3600 seconds of isothermal crystallization (hot stage temperature = 45°C), 50x (image dimensions: 175 × 131.2 *μ*m).

The development of crystalline structures at 45°C is shown in the optical micrographs reported in Figures 4, 5, and 6. On observing the morphology evolution by the polarized optical microscope, it was possible to measure the evolution of birefringence. Birefringence was then normalized according to the following equation:

(2) $$\begin{array}{c} \alpha_{\text{POM}}\left(t\right)=\frac{\Delta n\left(t\right)-\Delta n_{0}}{\Delta n_{\infty }-\Delta n_{0}}, \end{array}$$

in which Δ*n* is the measured birefringence at a given time, Δ*n*_0_ is the birefringence at the start of the isothermal test (which is nearly zero), and Δ*n*_*∞*_ is the birefringence at the end of crystallization (when a plateau is reached). Being birefringence essentially proportional to crystallinity, *α*_POM_(*t*) defined by (2) can be used to monitor the evolution of crystallization in thin samples.

**Figure 7:**
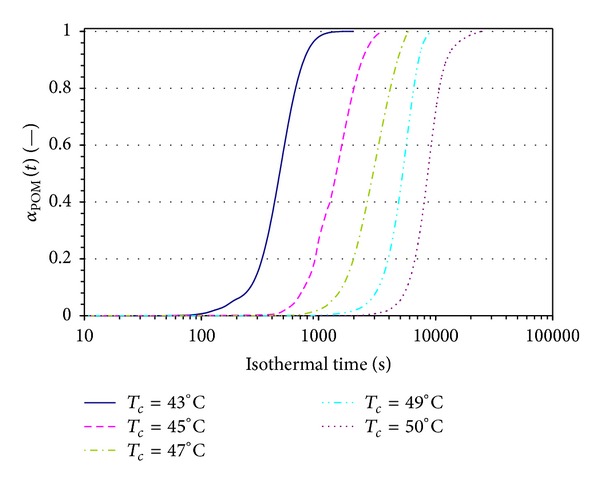
Evolution of *α*_POM_ (defined by (2)) in isothermal crystallization.

In Figure 7, the evolution of *α*_POM_ is plotted as a function of time for a series of isothermal tests similar to those presented in Figure 2. As the crystallization temperature increases, the sigmoidal curves are shifted parallel to each other on a linear-log scale. At the highest temperature used here, the time to reach the birefringence plateau has been increased by an order of magnitude.

It is important to point out that evolution intensity expressed by (2) does not provide a direct measure of the degree of crystallinity. Polymer crystallizing systems consist of many birefringent spots, arranged in the light path either side by side or in series. Ziabicki [20] argued that single-crystal formula (2) should be applied only to very dilute systems and/or very thin samples, in which probability of the appearance of more than one crystal in the light path is negligible. On the other hand, Binsbergen [21] developed a model of stacks of plates with small optical retardation, claiming applicability of the single-crystal formula to nondilute systems. More in general the depolarization ratio is not proportional to crystallinity alone but to crystallinity multiplied by a function of average plate dimensions [22]. However, assuming directly observable quantity *α*_POM_ as a crystallization characteristic per se, information about structure formation (induction time) and structural changes can be obtained.

### 3.3. Rheological Experiments

**Figure 8:**
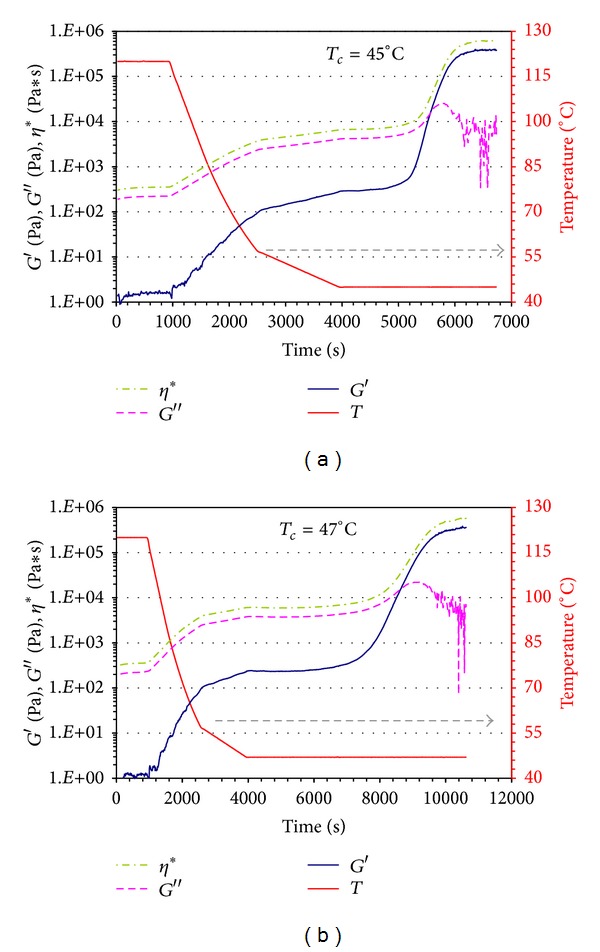
(a) Rheological measurements of the polycaprolactone crystallization at 45°C: storage modulus *G*′, loss modulus *G*′′, complex viscosity η*, and temperature measured simultaneously under constant stress of 100 Pa and constant frequency of 0.1 rad/sec. (b) Rheological measurements of the polycaprolactone crystallization at 47°C: storage modulus *G*′, loss modulus *G*′′, complex viscosity η*, and temperature measured simultaneously under constant stress of 100 Pa and constant frequency of 0.1 rad/sec.

In Figures 8(a) and 8(b) the evolution of the storage modulus (*G*′), the loss modulus (*G*′′) and the complex viscosity (η*) versus time during both the cooling (after the annealing process at *T* = 120°C) and the isothermal crystallization is reported together with the corresponding temperature profiles for two different quiescent crystallization tests.

The experiment shows that both moduli (*G*′ and *G*′′) are susceptible to structural changes in the polymer. As an example, *G*′ after a first increase during the cooling step, which can be ascribed to the effect of temperature, shows a plateau at *T* = *T*_*c*_, followed by a second increase representative of the crystallization process. Finally, a second plateau is found when crystallinity reaches a maximum value.

From the rheological experiment, the induction time for the isothermal crystallization can be detected at the time when the storage modulus abruptly increases. The transformed crystallized fraction *α*_RHEO_(*t*) has been estimated from the time dependent *G*′(*t*) values according to the following equation [23]:

(3) $$\begin{array}{c} \alpha_{\text{RHEO}}\left(t\right)=\frac{G_{}^{\prime }\left(t\right)-G_{0}^{\prime }}{G_{\infty }^{\prime }\left(t\right)-G_{0}^{\prime }}, \end{array}$$

where *G*_*o*_′, *G*′(*t*), and *G*_*∞*_′ are the melt stiffness values at time 0, *t*, and infinity, respectively.

**Figure 9:**
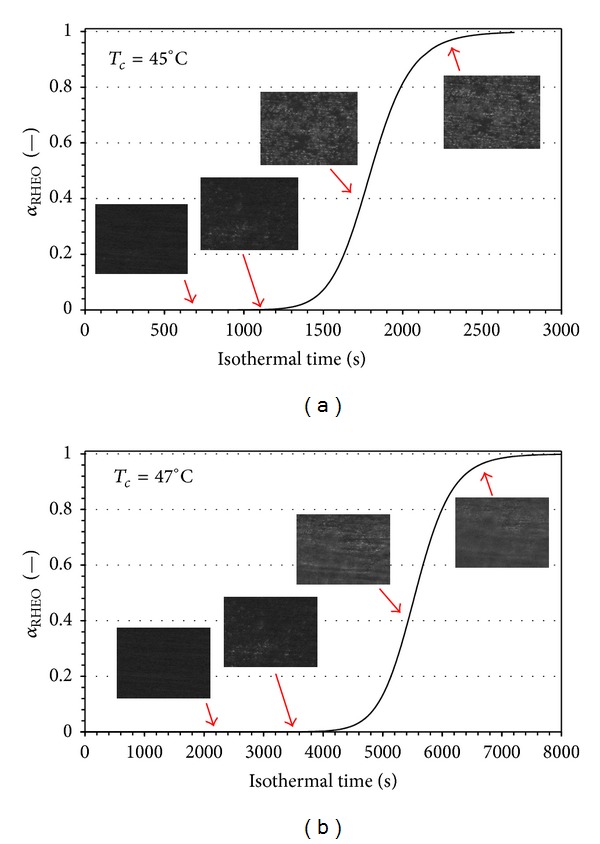
(a) Comparison between rheological evolution and optical morphology during isothermal crystallization of PCL at *T* = 45°C. (b) Comparison between rheological evolution and optical morphology during isothermal crystallization of PCL at *T* = 47°C.

The results for the structure development during the isothermal quiescent crystallization have been obtained at different crystallization temperatures. In Figures 9(a) and 9(b) the evolution of *α*_RHEO_ to the isothermal crystallization at 45°C and 47°C, respectively, is reported.

The time scale starts at the end of the cooling step for both the rheological and the morphological tests. The times read from the figures are, then, directly related to the evolution of the crystallization in isothermal condition.

The morphological investigation shows that spherulites develop and grow until the impingement occurs. As expected, the number of nuclei increases as the isothermal crystallization temperature decreases. What is interesting is the comparison between the structure development and the *G*′ trend (Figures 9(a) and 9(b)). Indeed, in all the cases it has been observed that spherulites appear and grow when the storage modulus is still constant at the first plateau value (representative of *G*′ for the undercooled melt at *T*_*c*_). This result is in agreement with the results reported by Acierno et al. [24], Bove and Nobile [25], and Pogodina et al. [26]. The increase in *G*′ during time after the impingement is still related to the crystallization process itself.

### 3.4. AFM Experiments

The same thermal protocol adopted before was replicated during AFM measurements. In that case, however, the recording of the data starts in parallel with the second cooling step (*T* < 60°C). In contrast to that observed with the calorimetric experiments, the first signal of structures formation was observed during the second cooling step, at temperatures just below 50°C. For that, it was impossible to carry out isothermal AFM tests at temperature lower than 50°C.

The evolution of the samples surface morphology was recoded in terms of distance from a reference position (height) and the characteristic oscillation (amplitude and phase) of the AFM cantilever during the observation times. The three acquired signals are strictly related to the structure formation on the sample surface during the crystallization phenomena. However, in our case, the amplitude signal was found the most sensible to the change in surface morphology.

**Figure 10:**
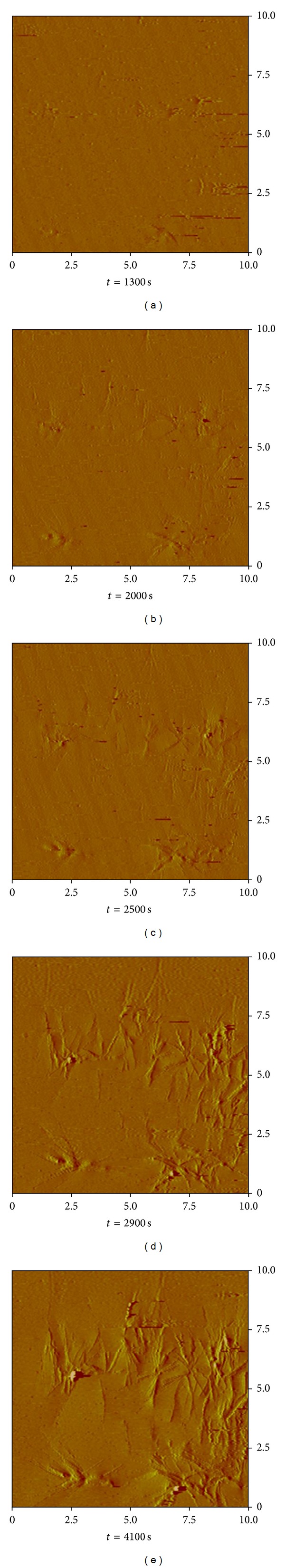
AFM amplitude images on a square area with sides of 10 *μ*m (*T*_*c*_ = 50°C).

For the present analysis, two different tests were considered: one with an observed square area with a side of 10 *μ*m and another with wider area having a side of 30 *μ*m. As shown in Figure 10, some evidence of crystalline structures appears on the surface after about 1500 s in isothermal conditions.

The crystalline structure development was followed by AFM while growing and it was found that the growth front propagates by the advancement of primary arms, with the regions between the arms filled in by secondary arms growing behind the primary front [27].

During the isothermal step new crystal aggregates were not formed but just the development of the previously formed structures was evident. This observation is coherent with the hypothesis of nucleation dependence on temperature and thus during the isothermal step no new stable nuclei are formed.

The development of new crystalline structures on the sample surface was followed by means of the evolution of the average value of the amplitude signal, $\overset{-}{A}(t)$, during the whole test.

In particular, by assuming as initial value of the averaged amplitude $\left({\overset{-}{A}}_{0}\right)$ the value that $\overset{-}{A}(t)$ assumes at starting crystallization time, the variations of the averaged amplitude for each time $\left(\overset{-}{A}(t)-{\overset{-}{A}}_{0}\right)$ divided by the total variation, recorded during the whole test, can be transformed into a variation index (*α*_AFM_(*t*)):

(4) $$\begin{array}{c} \alpha_{\text{AFM}}\left(t\right)=\frac{\int_{0}^{t}\left(\overset{-}{A}\left(t\right)-{\overset{-}{A}}_{0}\right)dt}{\int_{0}^{\infty }\left(\overset{-}{A}\left(t\right)-{\overset{-}{A}}_{0}\right)dt}. \end{array}$$

**Figure 11:**
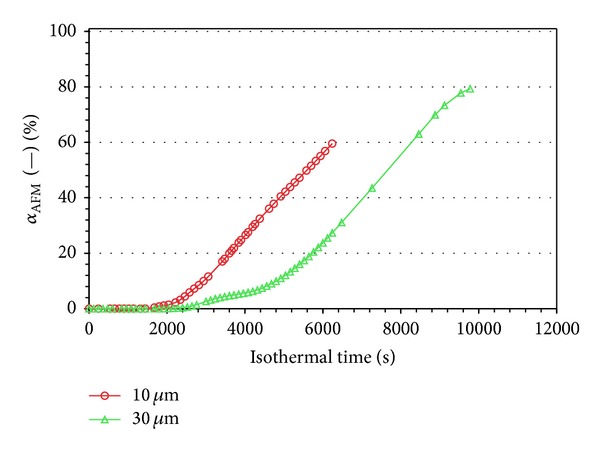
Evolution of averaged amplitude of AFM test on a square area with sides of 10 *μ*m and 30 *μ*m.

In Figure 11 is reported, for both tests conditions, the evolution of *α*_AFM_(*t*) as function of the isothermal crystallization time.

At early stage of the crystallization process, a small part of the investigated surface is covered by crystalline structure, so the variations of the averaged amplitude signal are very low. As the crystallization proceeds, the variations of the averaged amplitude become larger until a large part of the surface is covered by the crystalline structure and therefore the variations of the averaged amplitude are constant.

**Figure 12:**
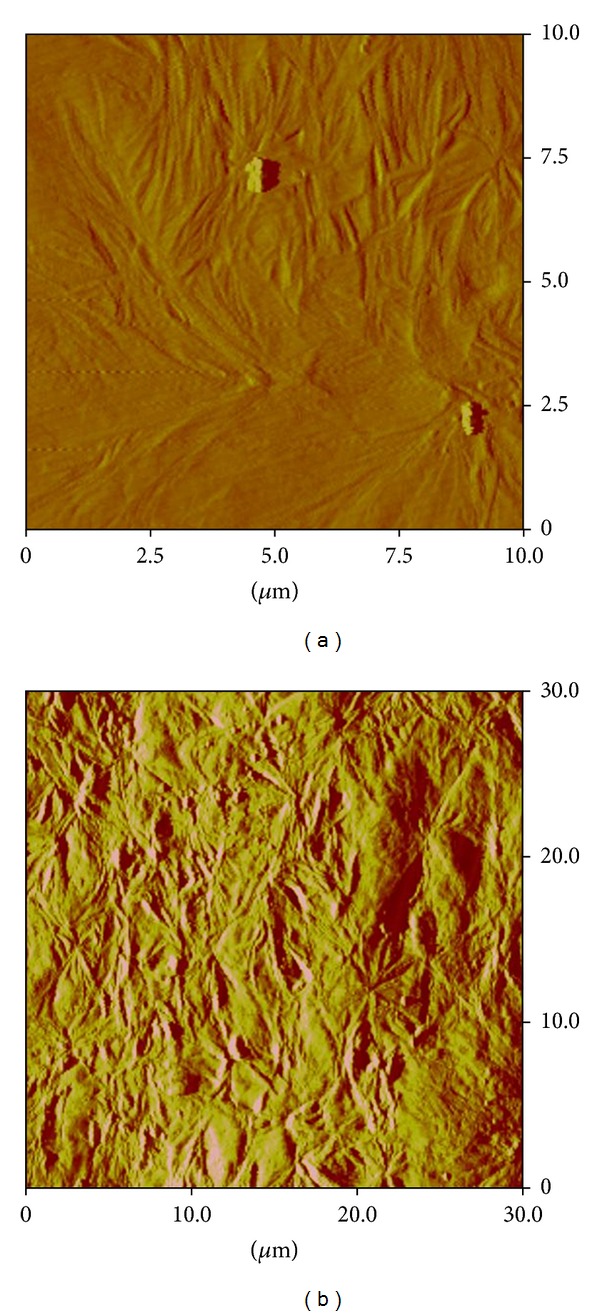
AFM amplitude images on different areas after complete crystallization.

As expected, even on the same sample, the morphology appears very different by changing the surface analyzed. This is clearly shown in Figure 12, where the same sample shows a different texture by changing the scanned area.

## Discussions

**Figure 13:**
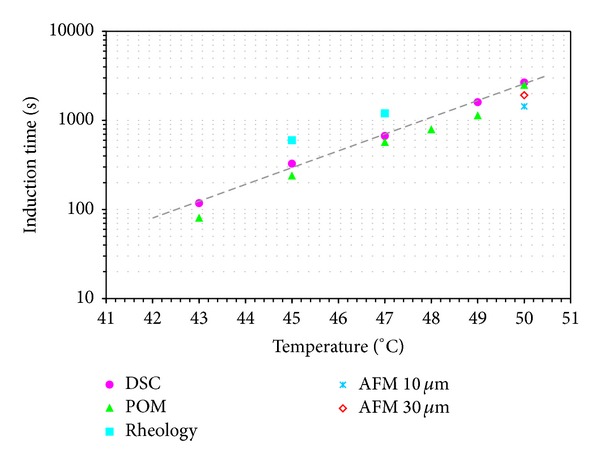
Induction time recorder by the different techniques. The dashed line represents a guide for eyes.

All the techniques adopted in this work were analysed in such a way to obtain sigmoidal curves representative of the evolution of the crystallization. From all the curves, it was possible to identify an induction time as the time at which the measured signal deviates from the value that it presents at the start of the isothermal step when the sample is still molten, without any crystallinity. A comparison between the results obtained with the different techniques presented is shown in Figure 13.

Results clearly reveal that the induction time found on the entire range of crystallization is dependent on the technique used. As expected, the observation scale plays an important role in its determination. Rheology appears to be the least sensitive technique, probably because in order to measure an increase in rheological parameters, an interaction among crystals is necessary. On the contrary, AFM tests that are able to resolve the surface sample at dimensions comparable to the primary nuclei can detect crystallization at initial stages.

## 5. Conclusions

The morphological development taking place during the crystallization of polycaprolactone (PCL) was studied using several techniques.

Results show that the induction time is dependent on the technique used. As expected, the observation scale plays an important role in its determination. In particular, despite its limitations, the AFM that is able to resolve the surface sample at dimensions comparable to the primary nuclei is able to detect crystallization at initial stages. On the contrary, rheology appears to be the least sensitive technique, probably because in order to measure an increase in rheological parameters an interaction among crystals is necessary. DSC and POM techniques supply closer results. It suggests that the definition of induction time in the polymer crystallization is a vague concept that, in any case, requires the definition of the technique used for its characterization.
